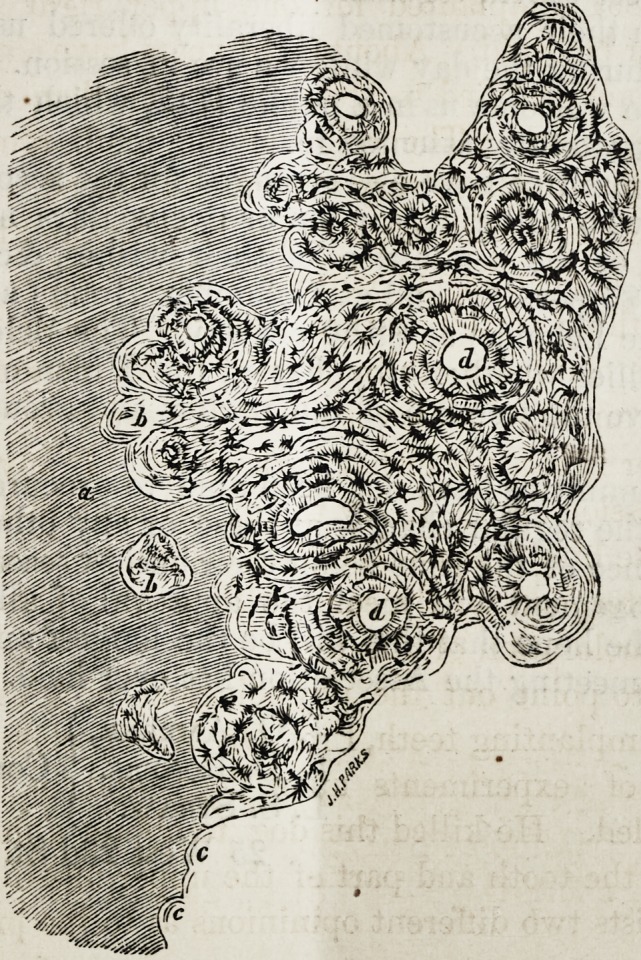# Replantation, Transplantation and Implantation

**Published:** 1869-07

**Authors:** O. Salomon


### ARTICLE IV.

### *Replantation, Transplantation and Implantation.*

**Translated from the German for the American Journal of Dental Science**

By Mr. O. Salomon.

Dr. Mitscherlich, Professor of Surgery in the University of Berlin, has written a Monograph upon this subject, and Dr. Suersen of Berlin mentioned something on the same subject at the annual meeting of the German Dentists.

In the first place to prevent misunderstanding, I will explain the difference between Replantation, Transplantation and Implantation. Replantation is to replace a tooth, after extraction, in the same alveola. Transplantation is the placing of a freshly extracted tooth from the mouth of one person into that of another. Implantation is the insertion of an old and dead tooth. Replantation and transplantation have been in use for a long time. The first successful implantation was performed by Prof. Dr. Mitscherlich who has also written a scientific paper upon the method of performing it.

Professor Dr. Mitscherlich presented to the Society of Medicine of Berlin (North Germany) a number of such patients. I have taken occasion to examine one of these patients before leaving Berlin, in order to be able to speak understandingly upon the "*status praesens.*" The case was that of a young lady, for whom Prof. Dr. M. on the 2nd of March, 1861, implanted for one upper right cuspid, a second lower bicuspid. About eight years have elapsed and this tooth I am gratified to state, is firmer than any other tooth in her mouth. The color of the tooth remains unchanged, a circumstance very uncommon indeed. The soft parts around the tooth are perfectly healthy, and it has not the slightest appearance of chronic inflammation, suppuration, or anything of this nature. In the same mouth there is another implanted tooth by Prof. Dr. M. inserted a little later, the firmness of which is about the same as the first, but the color is of a somewhat dark violet. If any member of the profession desires to try the experiment of implantation, I advise him to open the pulp cavity, remove the soft, organic matter, and fill the same with gold. This will prevent the discoloration of the tooth.

In order to point out the physiological progress of this method of implanting teeth, Prof. Dr. Mitscherlich has tried a number of experiments upon dogs, and in only one case succeeded. He killed this dog to obtain a microscopical view of the tooth and part of the upper maxilla.

There exists two different opininions as to the progress of replanted teeth ; the one is that the tooth has an active tendency to grow into vitality, the other is that it plays but a passive part. The first is not impossible as may be ascertained by examining a preparation of this nature in the anatomical museum at Bonn. As to implanted teeth, they having been already dead, vital action is impossible.

The progress of the latter is, according to Prof. Dr. M.'s experiments, as follows : After the extraction of the roots the periosteum of the alveola swells slightly and forms an exudation which surrounds the roots of the implanted tooth, and if the tooth is not artificially retained it will eject it ; at the same time with the process of exudation and undoubtedly caused by it, the cementum and dentine of the implanted root are re-absorbed in some places. In the resorbed places, the exudation grows and ossifies after a time and the tooth is thus let into place. The success of this operation depends upon the presence of the vital process of the alveola.

This cut represents a vertical section of a dead tooth implanted in a dog, and of the maxilla adjoining magnified 120 times.

*a*—Dentine.

*b*—New formed bone growing into the dentine.

*c*—Cavity of the reserved dentine.

*d*—Haversian Canals.

### CORRESPONDENCE.

## Figures and Tables

**Figure f1:**